# Spatially Resolved Experimental Modal Analysis on High-Speed Composite Rotors Using a Non-Contact, Non-Rotating Sensor

**DOI:** 10.3390/s21144705

**Published:** 2021-07-09

**Authors:** Julian Lich, Tino Wollmann, Angelos Filippatos, Maik Gude, Juergen Czarske, Robert Kuschmierz

**Affiliations:** 1 Laboratory for Measurement and Sensor System Technique, TU Dresden, Helmholzstr. 18, 01069 Dresden, Germany; juergen.czarske@tu-dresden.de (J.C.); robert.kuschmierz@tu-dresden.de (R.K.); 2 Institute of Lightweight Engineering and Polymer Technology, TU Dresden, Holbeinstr. 3, 01307 Dresden, Germany; tino.wollmann@tu-dresden.de (T.W.); angelos.filippatos@tu-dresden.de (A.F.); maik.gude@tu-dresden.de (M.G.); 3 Lab Dresden Center for Intelligent Materials (DCIM), TU Dresden, 01069 Dresden, Germany

**Keywords:** experimental modal analysis, modal testing, rotating frame, stationary frame, rotor dynamics, rotating structures, optical measurement, diffraction grating sensor

## Abstract

Due to their lightweight properties, fiber-reinforced composites are well suited for large and fast rotating structures, such as fan blades in turbomachines. To investigate rotor safety and performance, in situ measurements of the structural dynamic behaviour must be performed during rotating conditions. An approach to measuring spatially resolved vibration responses of a rotating structure with a non-contact, non-rotating sensor is investigated here. The resulting spectra can be assigned to specific locations on the structure and have similar properties to the spectra measured with co-rotating sensors, such as strain gauges. The sampling frequency is increased by performing consecutive measurements with a constant excitation function and varying time delays. The method allows for a paradigm shift to unambiguous identification of natural frequencies and mode shapes with arbitrary rotor shapes and excitation functions without the need for co-rotating sensors. Deflection measurements on a glass fiber-reinforced polymer disk were performed with a diffraction grating-based sensor system at 40 measurement points with an uncertainty below 15 μrad and a commercial triangulation sensor at 200 measurement points at surface speeds up to 300 m/s. A rotation-induced increase of two natural frequencies was measured, and their mode shapes were derived at the corresponding rotational speeds. A strain gauge was used for validation.

## 1. Introduction

Rotor dynamics play a crucial role in many industries, such as the automotive, aerospace and energy sectors [1,2,3]. Knowing, understanding and predicting their dynamic properties is necessary for designing and operating rotating systems, such as turbines, gears, pumps and ventilators, with high safety, performance and efficiency [4]. In order to avoid wear or catastrophic failure of a rotating system, its vibration modes should be properly damped and their natural frequency should not coincide with the rotational speed. Especially at fast rotating structures, such as turbine fan blades, the rotational speed has a significant influence on the spectral properties of the rotor. Additionally, factors such as temperature and damages can severely influence the spectral properties of rotors. Damping, nonlinearities, manufacturing uncertainties and fluctuating boundary conditions can lead to high prediction uncertainties of purely numerical models. Therefore, in situ measurements are required [5].

To perform an experimental modal analysis of rotating structures, the structures must be mechanically excited with a known excitation force function. The force can be induced by piezoelectric actuators or electromagnets. Another possibility for mechanical excitation is to store potential energy, e.g., internal deformation, within the structure itself and to then convert it to kinetic energy [6].

The vibration response can be measured from the rotating frame or the stationary frame. During a measurement from the rotating frame, the sensor rotates with the structure. During a measurement from the stationary frame, the sensor does not rotate with the rotor, so that there is relative movement between rotor and sensor.

Rotating frame spectra are acquired at specific measurement points by applying sensors to the rotor. Typically, accelerometers [7,8], piezoelectric transducers [9], strain gauges [8,10] or fibre Bragg gratings [11] can be deployed. Such local sensors offer high temporal resolution and low measurement uncertainties. However, the spatial resolution is limited by the number of applied sensors and transmission channels [12]. Furthermore, the applied sensors and associated wiring can influence the structure dynamic behaviour of the specimen [13].

For non-contact measurements from the stationary frame, deflection or velocity sensors, such as triangulation, laser Doppler distance sensors [14,15], laser Doppler vibrometry [16,17,18] or non-optical sensors [19,20,21] can be used. Optical sensors can achieve high SNR, especially if the optical properties of the rotor surface are modified. This is the case with diffraction grating sensors (DGS), which are optically read out by a light beam and a telescope setup [22]. With such methods, the modal testing process can be performed noninvasively—if the excitation is induced externally [23]. Furthermore, high spatial resolution and therefore the assignment of shapes to high-order modes is achievable. However, in case of a continuous measurement from the stationary frame, the response spectrum results from the circumferential trajectory of the sensor. The spectrum has therefore inherently different characteristics than the single spot rotating frame spectra within the trajectory [24,25,26]. Furthermore, such stationary frame spectra are prone to ambiguities in mode determination, as both spatial information and vibration frequencies are encoded into the response spectra. Especially if excitations with more than one frequency at a time, such as when an impulse or random excitation, are used, such ambiguities can hardly be resolved. Furthermore, continuous measurements from the stationary frame might be prevented, e.g., by propeller-like structures or a limited amount of measurement points. Such circumstances can lead to additional aliasing effects.

Full field methods, such as 2D or 3D digital image correlation (DIC) or electronic speckle pattern interferometry (ESPI), can be used [13,27,28]. However, such methods suffer from low SNR, as DIC has a limited measurement resolution, especially at high surface speeds, and ESPI is prone to rigid body movement-induced speckle decorrelation.

Sensors from the stationary frame category as well as full field methods can be used combined with an optical derotator [18,29,30,31]. This allows for the acquisition of point-to-point rotating frame spectra with a stationary frame sensor. However, such systems are expensive, special test rigs with optical access are necessary, and the alignment of the rotational axes of the rotor and derotator is elaborate [32]. Furthermore, such systems can suffer from harmonics due to the rotational speed in the spectral response [33] and the numerical aperture is too small to effectively perform triangulation techniques for larger rotors.

In this paper, an approach—referred to as rotating frame measurement from the stationary frame (RSFM)—is investigated. RSFM allows us to measure point-to-point rotating frame response spectra, e.g., as measured by accelerometers and strain gauges on the rotor, with a non-contact sensor, such as a triangulation sensor. As a consequence, rotating frame sensors do not have to be placed on the rotor. As non-contact sensors additionally allow for high spatial resolution, the identification of high-order mode shapes is possible at arbitrary rotor forms and without aliasing ambiguities that arise from continuous stationary frame measurement.

The mentioned acquisition methods, including RSFM, are described in Section 2. In Section 3, RSFM is verified by resampling of the frequency response of a non-rotating rotor. Section 4 shows the results of a modal analysis of a fast rotating fiber-reinforced polymer disk at varying rotational speeds. Waterfall plots and mode shapes are derived. The measurements are performed with optically read out DGS on the rotor surface and a commercial triangulation sensor. A comparison to rotating frame spectra, which are acquired by a strain gauge on the rotor surface, is made for validation and a comparison between the sensors regarding the SNR is conducted. Additionally, the results of the triangulation sensor are used to perform a direct comparison between the stationary frame measurement (SFM) with RSFM.

## 2. Theory

### 2.1. Rotating Frame Measurement (RFM)

To perform experimental modal analysis on a rotating structure, it can be mechanically excited by a force f(t), which can be induced, e.g., by a piezo actuator. The vibration response $w_{r,P}\left(t\right)$ is then measured at multiple locations P, e.g., by accelerometers or strain gauges. By dividing the spectrum of the response $W_{r,P}\left(f\right)$ by the spectrum of the force F(f), the frequency response function (FRF) can be derived. Analogously to the sampling theorem [34], the mode shape in a certain direction can be determined by measuring the FRF at twice as many locations as the mode order *M* in the corresponding direction. Hence, due to the limited number of sensors or transmission channels, the identification of higher modes is limited.

### 2.2. Stationary Frame Measurement (SFM)

**Figure 1:**
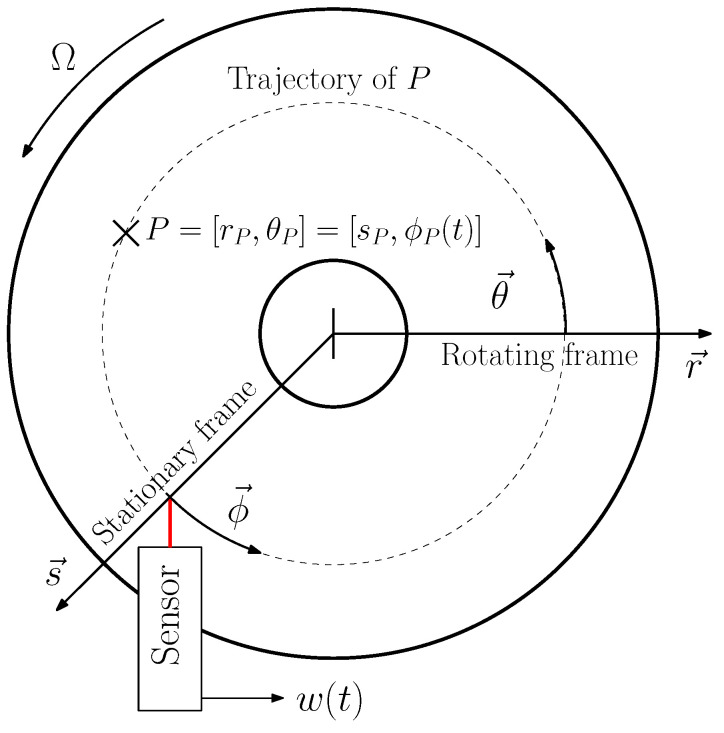
Sketch of a disk, rotating with Ω, and a sensor, fixed to stationary frame. Under the assumption of no eccentricity, the trajectory of a Point *P* on the disk is a circle so that only the angle $\phi_{p}$ is time dependent at Ω>0 Hz.

A sensor that is fixed to the stationary frame measures the response along the circumferential trajectory of a rotor (see Figure 1) if it rotates with the rotational speed Ω. The frequency spectrum $W_{s}\left(f\right)$ of the continuous sensor signal $w_{s}\left(t\right)$ therefore differs inherently from $W_{r,P}\left(f\right)$ generated with RFM.

If the rotor is a disk-like structure (The diametrical mode shapes of disk-like structures can be approximated to be purely sinusoidal and nodal circles can be neglected [26]. Vibration modes of other structures can contain multiple diametrical and circular components at once, which results in additional peaks in the stationary frame spectrum) in vacuum or air, and the peaks in $W_{r,P}\left(f\right)$ at every excited natural frequency $\omega_{M,r}$ are split into two peaks $\omega_{M,s\pm }$ each, according to [26]

(1) $$\omega_{M,s}=\omega_{M,r}\pm M\Omega ,$$

where *M* is the number of nodal diameters. For demonstration purposes, the deflection signals from a rotating flat disk that oscillates sinusoidally were simulated according to RFM and SFM. In the case of RFM, the measurement position *P* and thus the observation angle $\theta_{P}$ were declared constant. The RFM signal was simulated as

(2) $$w_{r,P}\left(t\right)=A_{0}\;cos\left(M\;\theta_{P}\right)\;cos\left(2\pi \;\omega \;t\right).$$

The amplitude $A_{0}$ was declared as 1 and unitless. The first cosine term describes the mode shape with *M* = 1. The second cosine term describes an oscillation with the natural frequency of ω = 250 Hz. The case of SFM was simulated by making the observation angle θ(t) change over time with a rate of 2πΩ, starting from θ(0) = 0 rad:

(3) $$w_{s}\left(t\right)=A_{0}\;cos\left(M\;2\pi \;\Omega \;t\right)\;cos\left(2\pi \;\omega \;t\right).$$

**Figure 2:**
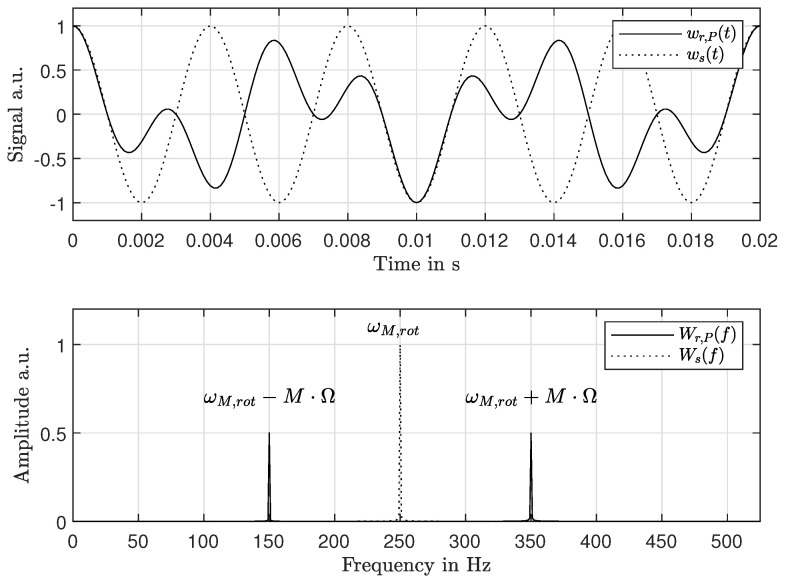
Simulation of the rotating frame (dashed) and stationary frame measurement (solid) at the outer radius of the disk, which vibrates with its first diametrical mode (*M* = 1) at 250 Hz and rotates with Ω = 100 Hz. *P* was set to a location of maximum amplitude.

The results for Ω = 100 Hz are plotted in Figure 2. Corresponding to Equation (1), the SFM signal $W_{r,P}\left(t\right)$ is split into two peaks in the frequency domain. As the position of these peaks depends on the number of nodal diameters *M*, the spectrum of the stationary frame measurement (SFM) can be used for mode decomposition. However, this peak multiplying spectrum also carries disadvantages. On the one hand, a direct comparison towards RFM is not possible. On the other hand, ambiguities of the spectrum can arise and prevent mode identification, especially if multiple natural frequencies are excited simultaneously.

### 2.3. Rotating Frame Measurement from the Stationary Frame (RSFM)

If the relative position between the rotor and sensor is known for w(t) at every point in time, it can be divided into small intervals, each of which correspond to a location P. By averaging every interval, a pseudo rotating frame signal $w_{P}\left(t\right)$ can be derived for every location *P* (see Figure 1). The sampling frequency for $w_{P}\left(t\right)$ is $\omega_{s}=\Omega$, as *P* passes the stationary frame sensor once per revolution. To cover all natural frequencies of interest, the spectral measurement range $\Delta \omega_{m}$ must be sufficiently large. The sampling theorem limits the spectral measurement range to $\Delta \omega_{m}=\Omega /2$, which, in most cases, is not sufficient to resolve the first natural frequency of the structure.

To artificially increase $\omega_{s}$ on *P*, $N_{c}$ sensor responses are measured with the same excitation function but varying time delays $\Delta t_{i}$ ($i=1\ldots N_{c}$) between the excitation start and the first sampling time.

$N_{c}=N_{sens}\cdot N_{meas}$ with $N_{sens}$ as the number of sensors along the trajectory of *P* and $N_{meas}$ as the number of measurement repetitions. In this paper, only a single sensor and thus $N_{c}=N_{meas}$ are considered. Under the assumption that Ω is constant, a matrix with the sampling times of all measurements can be formulated:

(4) $${\left(t_{ij}\right)}_{P}={\left(\begin{array}{c} \left(1,\ldots ,N_{r}\right)\cdot \frac{1}{\Omega }+\Delta t_{1}\\ \;\vdots \\ \;\left(1,\ldots ,N_{r}\right)\cdot \frac{1}{\Omega }+\Delta t_{N_{c}} \end{array}\right)}_{N_{c}\times N_{r}},$$

where $N_{r}$ is the number of rotor revolutions. The measurement values can be formulated in the same $N_{c}\times N_{r}$ structure analogously:

(5) $${\left(w_{ij}\right)}_{P}=w\left({\left(t_{ij}\right)}_{P}\right).$$

$\left(t_{ij}\right)$ and $\left(w_{ij}\right)$ are then combined with $(N_{c}\cdot N_{r})\times 1$—vectors ${\vec{t}}_{c,P}$ and ${\vec{w}}_{c,P}$—in a way that the time values are sorted in an ascending order and the measurement values undergo the same index changes as the time values. If the measurements and excitations are time invariant and noise free and there is no other excitation apart from the known source, all sampled measurement values captured on *P* from the stationary frame—and processed with RSFM—align with the continuous response viewed from the rotating frame $w_{r,P}$:

(6) $${\vec{w}}_{c,P}=w_{r,P}\left({\vec{t}}_{c,P}\right).$$

From now on, the special case of increasing $\Delta t_{i}$ with fixed intervals Δt, which are equally distributed within 1/Ω (uniform sampling), is assumed. (In reality, uniform sampling cannot be achieved due to trigger uncertainties and jitter of the rotational speed. However, the resulting non-uniformly sampled signal can be interpolated to a uniformly sampled signal without loss of quality as long as the average sampling rate is the same [35,36].)

(7) $$\Delta t_{i}=\left(i-1\right)\cdot \Delta t.$$

To reach or surpass a set sampling frequency $\omega_{s,set}$, the number of measurements is set to

(8) $$N_{c}=ceil\left(\omega_{s,set}/\Omega \right).$$

This leads to an effective sampling frequency and time intervals of

(9) $$\omega_{c}=1/\Delta t=\Omega \cdot N_{c}.$$

## 3. Verification of RSFM

**Figure 3:**
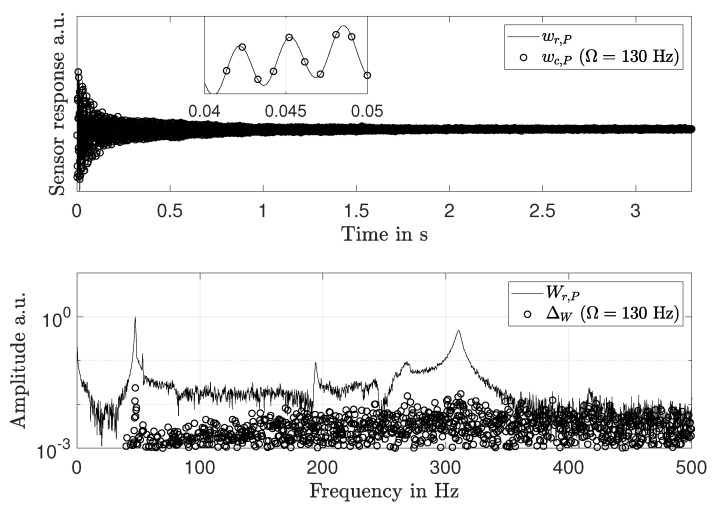
**Top**: rotating frame response $w_{r,P}$ and response $w_{c,P}$ generated with RSFM ($\omega_{s}$ = 1040 kHz) from simulated stationary frame sensor signals at Ω = 130Hz. **Bottom**: rotating frame response spectrum $W_{r,P}$ and measurement deviation $\Delta_{W}$ to RSFM spectrum $W_{c,P}$.

In order to verify this approach, a measurement is simulated. First, an impulse response $w_{r,P}$ for a single point *P* on a composite disk is generated. The response has a bandwidth of 500 Hz. A rotational frequency Ω = 130 Hz is assumed. Since *P* passes the sensor once per revolution, $w_{r,P}$ is downsampled to 130 Hz. The measurement is repeated $N_{c}$ = 8 times with varying time delays $\Delta t_{i}$ and combined to a signal $w_{c,P}$ with a sampling rate of 1040 Hz. The time signals $w_{r,P}$ and $w_{c,P}$ are shown in Figure 3 (top). In Figure 3 (bottom), $W_{r,P}$ and the measurement deviation $\Delta_{w}$ to $W_{c,P}$ are shown. The mean deviation is 0.24% of the maximum value of $W_{r,P}$. Hence, RSFM allows to precisely recuperate rotating frame responses with a stationary frame sensor.

**Figure 4:**
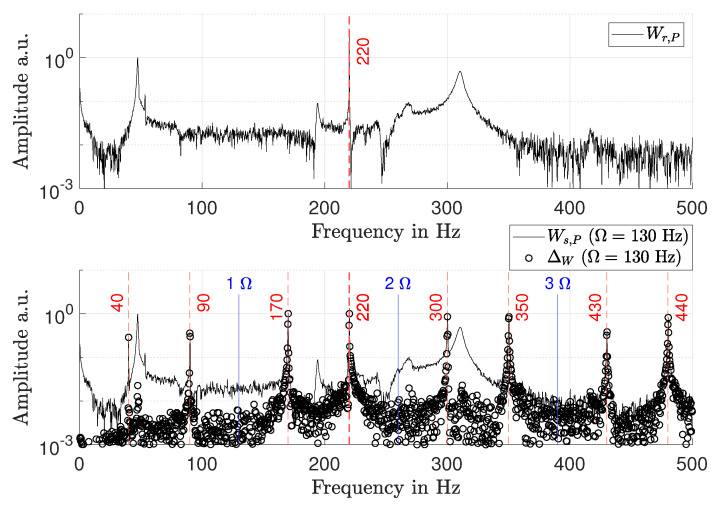
**Top**: rotating frame response spectrum $W_{r,P}$ with a superposed sine function at 220 Hz. **Bottom**: RSFM spectrum $W_{c,P}$ and measurement deviation $\Delta_{W}$ at Ω = 130 Hz. The added sine function at 220 Hz has a random phase at every consecutive measurement.

However, RSFM requires a repeatable rotor response for all consecutive measurements. While actuators allow for repeatable excitation, there can be other non-repeatable vibration sources, such as the drive of the test rig or air friction. The influence of such non-repeatable vibration sources on the measurement is simulated by adding a sine function to $w_{r,P}$ with a frequency of 220 Hz and a random phase for each of the $N_{c}$ = 8 measurements. Figure 4 (top) shows $W_{r,P}$, which is superposed with the sine function. In Figure 4 (bottom), the effect on $W_{c,P}$ and $\Delta_{w}$ is shown. The superposed out-of-phase signal causes aliasing around multiples of the rotational speed Ω, which results in a significant increase of the measurement deviation. Especially if broad band out-of-phase excitation sources are present or if the aliased peaks coincide with in-phase peaks, ambiguities in mode identification can arise. Therefore, RSFM requires test environments that keep additional vibrations at a minimum.

## 4. Experimental Results

### 4.1. Setup and Measurement Procedure

**Figure 5:**
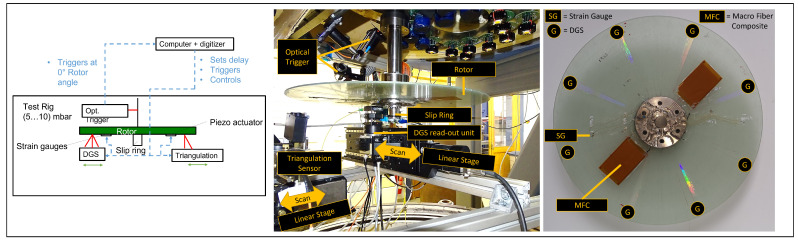
**Left**: functional sketch of setup. **Center**: photograph of the setup. **Right**: photograph of the rotor.

To validate the method, a glass fiber-reinforced polymer disk with a diameter of 500 mm was used as test specimen and accelerated inside a test rig, as seen in Figure 5 (right). During the measurements, the pressure inside the test rig was kept below 10 mbar to avoid rotor heating from air friction. Piezoelectric Macro Fiber Composite (MFC) patches from SMART MATERIALS were applied onto the rotor surface to excite the rotor. The excitation signals were generated with an NI USB-6212 digitiser and transmitted over an amplifier and a slip ring to the MFC. A WAYCON LAM-10 triangulation sensor as well as a DGS [22] were used to measure the rotor deflection according to RSFM. Diffraction grating sensors (DGS) were placed at eight equally distributed angles, each over the whole rotor radius. The triangulation sensor and the optical read-out unit for the DGS were placed on linear stages to scan the rotor along its radius. A rotor angle dependent optical trigger was used to synchronise the excitation and measurement. The rotor was excited with a linear sine sweep with f = (30…470) Hz over t = 3.3 s. Varying time delays $\Delta t_{i}$ between trigger signal and excitation were set to achieve a measurement rate of ≈ 1 kHz. Next to the MFC, a semiconductor strain gauge (*k* = 116, 400 Ohm, quarter-bridge, 5 V supply voltage) was applied to the rotor. A slip ring was used for data transfer.

The FRF matrix of the disk was measured at seven different rotational frequencies ranging from 0 Hz to 180 Hz. For each rotational frequency, five radii were scanned by the triangulation sensor and the DGS-read-out-unit. In total, the response spectra of 38 locations were acquired with DGS, while a continuous measurement over all angles took place with the triangulation sensor. For the triangulation sensor, the angular resolution was set to 4${}^{\circ }$, which corresponds to the angular extent of the DGS. For every location, the spectrum was calculated according to the RSFM method. Uniform sampling was achieved by an interpolation of the combined signals, both for DGS and triangulation.

### 4.2. Response Spectra and Mode Shapes

**Figure 6:**
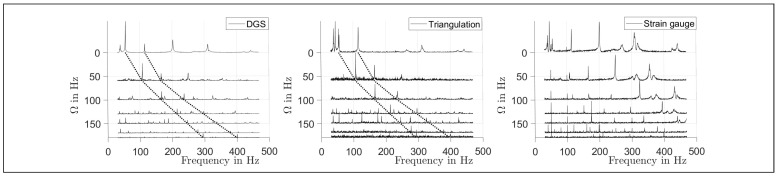
Waterfall plots of surface tilt measurement with the DGS, axial displacement measurement with triangulation and strain gauge at one location on the rotor. For the DGS and the triangulation sensor, an FRF of a location at *r* = 237 mm is shown. The strain gauge was placed at *R* = 170 mm. The dotted lines indicate the peaks of the second and third tangential modes, as plotted in Figure 7.

**Figure 7:**
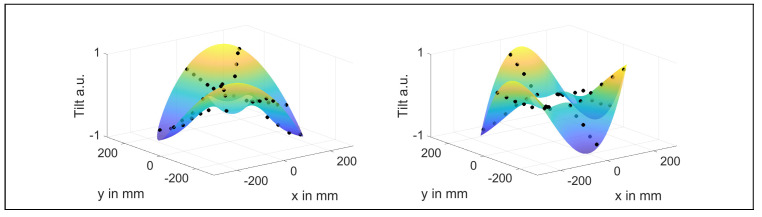
Complex amplitudes measured by the DGS and a fit to the theoretical mode shapes. **Left**: second tangential mode. **Right**: third tangential mode.

In Figure 6, the FRFs measured by the DGS, the triangulation sensor and the strain gauge are shown for various rotational speeds. In the case of the DGS, the rotor tilt α was evaluated. The dotted lines indicate the course of the frequencies of the modes *M* = 2 and 3. The mode numbers *M* were determined by fitting the theoretical mode shapes into the spatially resolved complex amplitudes of the FRF at the corresponding peak frequency, as can be seen in Figure 7. For the strain gauge, no mode identification was possible due to a lack of spatial resolution.

Figure 6 shows that the modal frequencies increase significantly from 55.7 Hz to 294.3 Hz (*M* = 2) and from 116.1 Hz to 399.9 Hz (*M* = 3) over the range of Ω = 0 Hz to 180 Hz, which is assumed to result from a stiffening of the rotor due to centripetal forces that were induced by the rotation [37].

### 4.3. Measurement Uncertainty

**Figure 8:**
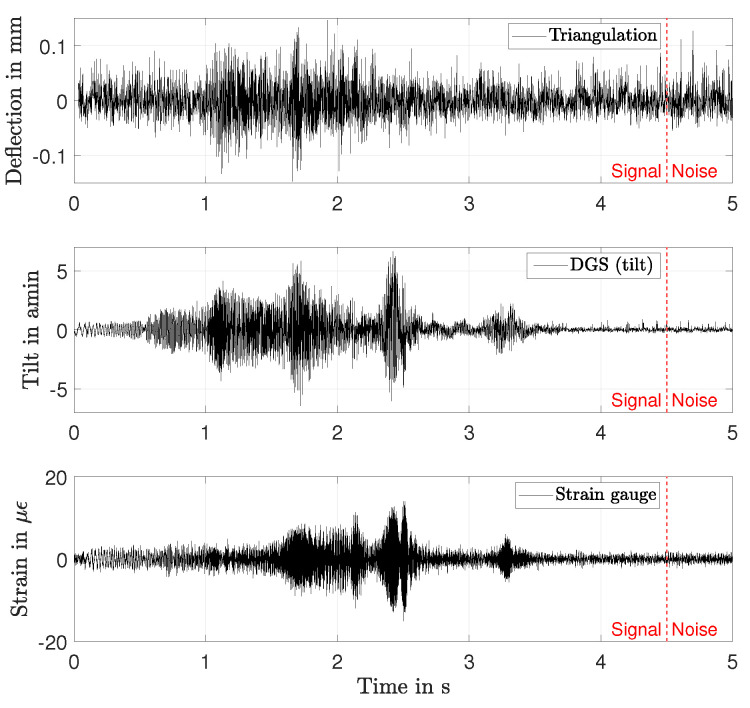
Sensor responses at one location at *R* = 170 mm and Ω = 60 Hz.

A comparison of the three applied sensors regarding their signal-to-noise-ratio (SNR) was conducted. An example of the individual sensor responses at one location at Ω = 60 Hz is given in Figure 8. The signals are bandpass filtered between (30…500) Hz. As marked with the dashed vertical line, the signals are split into a signal period $T_{ex}$ from (0…4.5) s, in which the excitation (0 s…3.3 s) and the swing-out (3.3 s…4.5 s) took place. $T_{ex}$ was followed by a noise period $T_{n}$ from 4.5 s, in which no excitation from the MFC took place. Hence, every deviation of the measurement signals from zero during $T_{n}$ is a consequence of sensor noise or an excitation from out-of-phase sources, such as the gear of the test rig or air friction. To estimate the SNR, the average powers $P_{ex}$ and $P_{n}$ within $T_{ex}$ and $T_{n}$, respectively, are used:

(10) $$SNR=\frac{P_{ex}-P_{n}}{P_{n}}.$$

**Figure 9:**
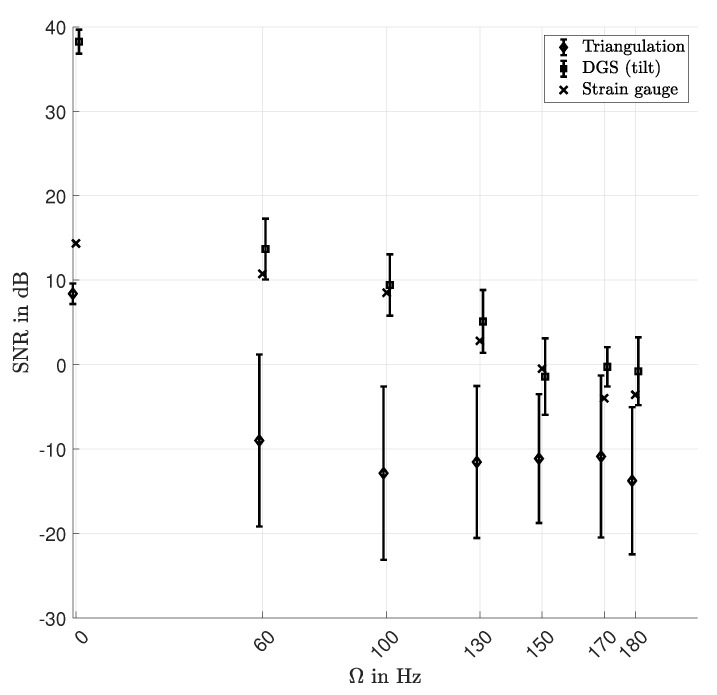
Median SNR and standard deviations (errorbars) over all measured angular locations at *R* = 170 mm.

The SNR were determined at 90 rotor angles for the triangulation sensor, at eight angles with the DGS an at one angle with the strain gauge. The median values and the standard deviations (triangulation and DGS) are plotted in Figure 9. The SNR for the DGS is the highest at Ω = 0 Hz and similar to the SNR of the strain gauge over all other rotational speeds. The SNR of the strain gauge was mostly limited by cross sensitivities to the power grid frequency and quantisation noise. The low SNR of the triangulation sensor results from the non-optimal optical surface properties of the translucent rotor. The SNR of all sensors tend to decrease with increasing Ω, which is assumed to be caused by a reduction of mode amplitudes due to an increase of the rotor stiffness and less integration time per location in case of the DGS and triangulation sensor. Especially, the results of the DGS and strain gauge show that, up to Ω = 130 Hz, a major part of the signal power resulted from the MFC excitation. At higher rotational speeds, the out-of-phase excitation forces dominated the signal. This can also be observed in Figure 6, where the peaks of the second and third modes become smaller for Ω > 130 Hz than their adjacent lobes, which are assumed to result from aliasing.

### 4.4. Comparison between SFM and RSFM on a Disk-Like Structure

**Figure 10:**
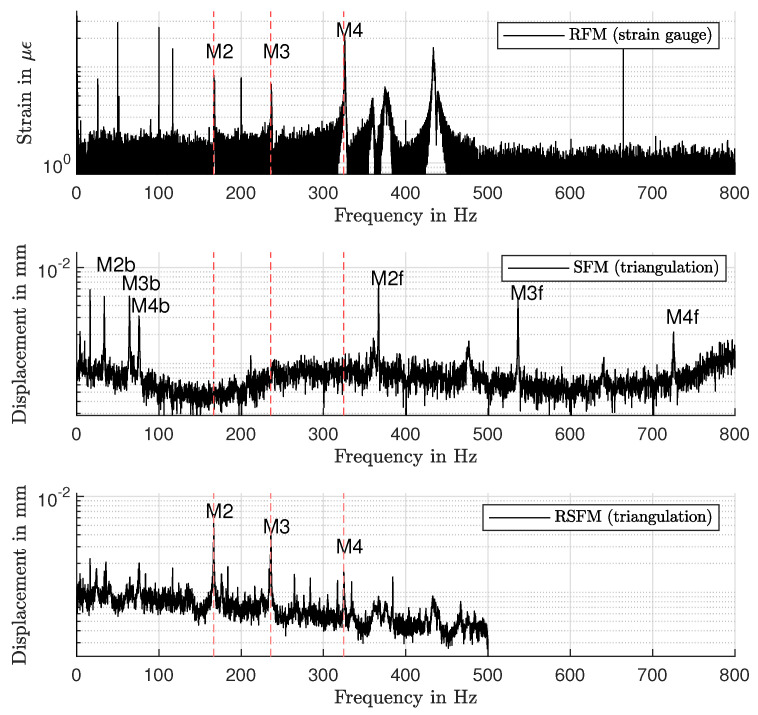
Measured spectra at Ω = 100 Hz. **Top**: RFM spectrum measured with strain gauge at *R* = 170 mm. Middle, **bottom**: SFM and RSFM spectra at *R* = 237 mm.

The results of the triangulation sensor at *R* = 237 mm were used. To evaluate the vibration induced spectral properties with SFM, the mean height profile of the surface was subtracted from the measured data. Additionally, an RFM spectrum was measured by a strain gauge at *R* = 170 mm on a single location for validation purposes. For RSFM, consecutive measurements per rotational speed were performed to generate 90 spectra (4° angular resolution) with the required bandwidth $\Delta \omega_{m}$ = 500 Hz. The median spectrum is shown in Figure 10. The results of SFM were generated with the same sensor data and show the median spectrum over $N_{c}$ measurements.

In the SFM spectrum, the peaks M2…M4 are split in forward (f) and backward (b) components, located symmetrically around the corresponding natural frequencies according to Equation (1). The backward components are aliased at 0 Hz. The spectra of RSFM and RFM show peaks at the natural frequencies of modes M2…M4 of the rotor. The additional peaks in the RFM spectrum are assumed to result from the sensitivity of the strain gauge towards in-plane deformations and disturbances from the supply voltage. The residual small lobes in the RSFM spectrum are assumed to result mainly from out-of-phase excitations in the test rig, as described in Section 3. The peak frequencies $\omega_{M2}\ldots \omega_{M4}$, measured with RFM, SFM and RSFM, are matched in Table 1, Table 2 and in agreement with the theory.

With SFM, spatial and temporal information are encoded in a single response spectrum, leading to ambiguities. Additional ambiguities arise due to possible aliasing around 0 Hz. Such ambiguities can be circumvented by a temporally resolved spectral analysis, e.g., with a wavelet transform. However, this requires special excitation functions. With RFM and RSFM, the natural frequencies are directly measurable and assignable to their mode shapes if measurements are performed at a sufficient amount of locations.

## 5. Conclusions and Outlook

Safe operation and the validation of numerical models for high-performance rotors require in situ measurements for modal analysis. To extract modal parameters, the rotating structures are excited and the response spectrum is measured. Continuous rotating frame measurements (RFM) offer high temporal resolution but lack spatial resolution. Continuous stationary frame measurements (SFM) offer high spatial resolution but suffer from low temporal resolution.

A new method called rotating frame measurement from the stationary frame (RSFM) was introduced. The rotor was consecutively excited with an arbitrary repeatable excitation function, which was induced after varying time delays with respect to the rotor angle. With RSFM, the unambiguous natural frequency determination of RFM and the high spatial resolution of SFM can be combined. The possible omission of RFM sensors and specimen preparation enables a noninvasive and fully featured experimental modal analysis of rotating structures. The method was verified by a simulation, and the influence of out-of-phase excitations was shown.

Modal analysis with RSFM was performed on a rotating fiber-reinforced polymer disk. Diffraction grating sensors (DGS) [22] and an optical triangulation sensor were used for the measurements. Response spectra and mode shapes were derived for a variety of rotational speeds up to 180 Hz. Additionally, RFM was conducted with a strain gauge for validation. The DGS and the strain gauge showed the highest signal-to-noise ratio. The measurement results show a significant increase in the natural frequencies with increasing rotor speed.

To distinguish between neighbouring modes in the frequency spectrum and to accurately and precisely determine modal parameters, increased measurement times are necessary. To reduce the overall measurement time, the method can be adapted to employing multiple sensors at varying rotor angles simultaneously.

RSFM can be used to perform fast, cost-effective and fully featured modal analyses on rotating structures, such as turbofans, in compact test rigs. No specimen preparation or elaborate alignment procedures, e.g., for Scanning Laser Doppler Vibrometry, are necessary. Therefore, RSFM is well suited for in-line assessments between rotor manufacturing and final assembly.

## Figures and Tables

**Table 1:** Rotating frame natural frequencies measured with RFM ($\omega_{RFM}$) and RSFM ($\omega_{RSFM}$). The rotating frame natural frequencies are compared to the backward and forward wave frequencies in the SFM spectrum $\omega_{SFM\mp }$. By matching the results, the natural frequencies can be assigned to their corresponding modes/number of nodal diameters *M*.

Name, Note	$\omega_{\mathrm{RFM}}$ in Hz	$\omega_{\mathrm{RSFM}}$ in Hz	M·Ω in Hz	$\omega_{\mathrm{RSFM}}\mp M\cdot \Omega$ in Hz	$\omega_{\mathrm{SFM}\mp }$ in Hz
M2, M=2	167.0	166.6	−200	−33.4	33.7
			+200	366.6	366.9
M3, M=3	236.7	236.0	−300	−64.0	64.2
			+300	536.0	536.4
M4, M=4	325.8	324.6	−400	−75.2	75.8
			+400	724.8	725.5

**Table 2:** Comparison between a rotating frame measurement (RFM), a stationary frame measurement (SFM) and a rotating frame measurement from the stationary frame (RSFM).

	RFM	SFM	RSFM
Spatial resolution	Low	High	High
Excitation function for	arbitrary	Single frequency	Repeatable,
unambiguous measurement		(e.g., sweep)	arbitrary
Unknown/out-of-phase	Add up in	Add up	Occur as aliasing
excitations	in spectrum	in spectrum	lobes in spectrum
Spectral	Peaks at natural	Decomposition	Peaks at natural
characteristics	frequencies	necessary	frequencies

## Data Availability

The data presented in this study is available upon request to the corresponding author.
